# Predicting nodal metastasis progression of oral tongue cancer using a hidden Markov model in MRI

**DOI:** 10.3389/fonc.2024.1360253

**Published:** 2024-06-06

**Authors:** Qiangqiang Gang, Jie Feng, Hans-Ulrich Kauczor, Ke Zhang

**Affiliations:** ^1^Department of Radiology, Southern Medical University Nanfang Hospital, Guangzhou, China; ^2^Department of Diagnostic and Interventional Radiology, Heidelberg University Hospital, Heidelberg, Germany

**Keywords:** oral cavity squamous cell carcinoma, lymph node metastases, artificial intelligence, hidden Markov model, level of metastatic lymph nodes

## Abstract

**Objectives:**

The presence of occult nodal metastases in patients with oral tongue squamous cell carcinomas (OTSCCs) has implications for treatment. More than 30% of patients will have occult nodal metastases, yet a considerable number of patients undergo unnecessary invasive neck dissection to confirm nodal status. In this work, we propose a probabilistic model for lymphatic metastatic spread that can quantify the risk of microscopic involvement at the lymph node level (LNL) given the location of macroscopic metastases and the tumor stage using the MRI method.

**Materials and methods:**

A total of 108 patients of OTSCCs were included in the study. A hidden Markov model (HMM) was used to compute the probabilities of transitions between states over time based on MRI. Learning of the transition probabilities was performed via Markov chain Monte Carlo sampling and was based on a dataset of OTSCC patients for whom involvement of individual LNLs was reported.

**Results:**

Our model found that the most common involvement was that of level I and level II, corresponding to a high probability of 𝑝b1 = 0.39 ± 0.05, 𝑝b2 = 0.53 ± 0.09; lymph node level I had metastasis, and the probability of metastasis in lymph node II was high (93.79%); lymph node level II had metastasis, and the probability of metastasis in lymph node III was small (7.88%). Lymph nodes progress faster in the early stage and slower in the late stage.

**Conclusion:**

An HMM can produce an algorithm that is able to predict nodal metastasis evolution in patients with OTSCCs by analyzing the macroscopic metastases observed in the upstream levels, and tumor category.

## Highlights

- Currently, lymphatic metastatic spread prediction for oral tongue SCCs (OTSCCs) is mostly based on the prevalence of nodal involvement.
- The risk of microscopic involvement in level III when level II was observed to harbor metastases increased. Similarly, the risk of microscopic involvement in level IV when patients with observed metastases in LNL II and III increased. Lymph node grade I–IV involvement occurred in early more than late stage in our data.
- This research illustrates the potential of the HMM-based model to personalize microscopic involvement risk based on the individual patient’s state of disease progression.

## Introduction

Currently, the combination of radiotherapy, chemotherapy, and intensity-modulated radiation therapy (IMRT) has substantially improved the locoregional control rates for patients with head and neck squamous cell carcinoma (HNSCC). An important aspect of these therapies is delineating the target tumor volume to ensure minimal effects in adjacent tissues. In HNSCC, target volumes are defined by three steps. First, the primary gross tumor volume (GTV-T) is delineated using CT, ^18^F-FDG-PET, or MRI (1). Secondly, tumor expansion was assessed under the microscope and GTV-T was extended to the primary clinical target tumor volume (CTV-T) (2). Third, an evaluation of lymph node metastases is assessed. Anatomically defined lymph node regions termed lymph node levels (LNL) divide the neck lymphatic system into groups. The definition of the selective clinical target nodal volume (CTV-N) is based on the included LNL. In recent years, there has been an international consensus on delineating LNLs (3–5). For example, Grégoire et al. (5) published an 88-page atlas detailing the anatomy of neck LNLs.

Clinical target volume 1 (CTV1) defines macroscopic disease, CTV2 defines microscopic high-risk disease, and CTV3 defines microscopic low-risk disease (6). Estimating the probability of microscopic involvement is challenging because it depends on several factors. Some of them include (1) the sensitivity and specificity of currently available imaging techniques, which affect the likelihood of an LNL metastasis despite negative imaging results (2), the location and staging of the primary tumor, influencing the likelihood of tumor spread to a given LNL, and (3) the local lymphatic spread of the primary tumor. To address these difficulties, oncologists have observed the patterns of lymphatic spread through clinical studies (7, 8), mostly reporting the proportions of specific LNL involvement based on the location and tumor staging of the primary tumor. Additionally, the sensitivity and specificity of imaging techniques have been studied by comparing the imaging performance and histopathology in surgical patients (5).

In head and neck cancers, most of them are SCCs; approximately 25% occur in the oral cavity, with the tongue being the most affected subsite (2). In this study, we propose to use a hidden Markov model (HMM) probability model to assess the spread of lymph node metastases and quantify the risk of microscopic infiltration at different LNLs given the macroscopic location and T-stage based on MRI. This research may allow for further personalized definitions of CTV-N based on the individual patient’s disease status using a time-series model suitable for MRI.

## Materials and methods

### Patient selection

A total of 131 patients with oral tongue squamous cell carcinomas (OTSCCs) who were admitted to our hospital between 10/10/2016 and 26/06/2023. Overall, 108 patients with OTSCCs who were treated with excision according to the criteria established by the National Comprehensive Cancer Network (NCCN) at Southern Medical University Nanfang Hospital were evaluated. The institutional review boards approved this retrospective study, and the requirement for informed consent was waived. Clinical characteristics, such as sex, age, areca nut history, and tumor, node, metastasis (TNM) stage, were recorded from the Hospital Information System (HIS) for all eligible patients. Patients’ serial MRI data were obtained from Picture Archiving and Communication Systems (PACS). The analysis of the lymphatic spread including levels I, II, III, and IV was performed for the diagnostic MRI imaging modalities including T1-weighted images, T2-weighted images, and contrast-enhanced T1-weighted images available for each patient. This was performed by two experienced radiologists by reviewing radiology reports together with the diagnostic images.

The inclusion criteria were as follows:

(1) OTSCCs were verified by pathology, i.e., before definitive radiotherapy with or without chemotherapy;
(2) patient with clinical tumor stage (T-stage) (T1–T4);
(3) lymph nodes that were clinically palpable or detectable on imaging at presentation;
(4) no previous/synchronous tumors;
(5) no previous neck surgery or “neck violation,” such as excisional nodal biopsy, fine-needle aspiration, or incisional biopsy with macroscopic/palpable tumor residual, allowed;
(6) dissection of at least three contiguous neck nodal levels
(7) lymph node surgery performed at Nanfang hospital, and
(8) neck specimen processed by surgical levels in the standard manner.

All eligible patients underwent baseline head and neck MRI scans before surgical treatment within 1 week. Diagnostic criteria for cervical lymph node metastasis were as follows: a smallest transverse diameter of more than 10 mm (5 mm–8 mm for retropharyngeal lymph nodes [level VIIA] and 12 mm–15 mm for upper jugular lymph nodes [level II]), central necrosis irrespective of the size, rounded rather than oval shape, loss of fatty hilum, visible peripheral extensions showing evidence of extracapsular spread, and the presence of more than three lymph nodes of size between 6 mm and 8 mm grouped (9). Two radiologists, each with over 8 years of experience and over 10 years of experience, independently evaluated the images, and in cases of disagreement for lymph node metastasis, assessments were resolved through consultation.

### Model training and validation

We modeled the patient’s state of metastatic lymphatic progression as a collection of hidden binary random variables that indicated the involvement of LNLs. In addition, each LNL was associated with observed binary random variables that indicated whether macroscopic metastases were detected. An HMM was used to compute the probabilities of transitions between states over time. Learning of the transition probabilities was performed via Markov chain Monte Carlo sampling and was based on the dataset of tongue cancer patients in whom involvement of individual LNLs was reported. We modeled the state of each LNL as a hidden or unobserved binary random variable, which was indicated via values 0 or 1 if an LNL was healthy or involved, respectively. This state indicates whether there is truly a tumor present in an LNL, including the presence of occult metastases for the involved state—motivating the term hidden or unobserved state. Every LNL can be diagnosed using MRI. The diagnosis was also modeled as a binary random variable—this time an observed one—taking on 0 for negative and 1 for positive.

**Figure 1:**
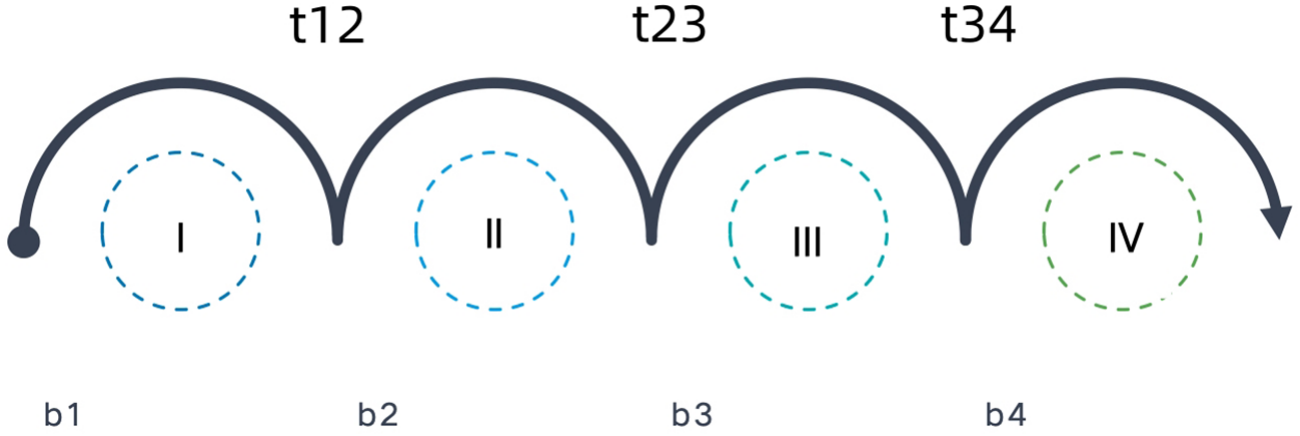
Annotated arcs depict the direction of lymphatic flow where the parameter next to it (b and t) represents the probability of metastatic spread.

The spread of the tumor through the lymphatic network is represented in this model by arcs directed to and between LNLs, as illustrated in **Figure 1**. Directed arcs from the primary tumor to an LNL represented the direct spread of tumor cells from the primary tumor (N0 status) to the LNL(N+ status). These arcs were associated with parameters b_*ν*_, which we called base probabilities, and which indicated the probability that the tumor could spread directly to LNL *ν*. When LNL *s* receives efferent lymphatics from LNL *r*, this was also represented by a directed arc from LNL *r* to *s* and *r*=pa (*s*), which is called a parent node of *s*. These arcs were associated with a transition probability *t_rs_* from *r* to *s*.

We used the parameters inferred from the early T-stage dataset and times prior regarding expectation of a patient being diagnosed later to estimate how risks of microscopic metastasis might increase over time. The parameters inferred from the late T-stage dataset were also analyzed.

In this work, we apply sensitivity and specificity parameters based on published literature and recent meta-analyses, i.e., these parameters were not learned from data. Specificity and sensitivity values for MRI diagnostic procedures in head and neck cancer were set as 0.63 and 0.81 (10). The data were split randomly into three equally large parts. Then, the model was trained on all three combinations of two of these thirds and compared with the remaining third to see if the results were still plausible. Bayesian network (BN) were used previously to model lymphatic spread (11), which is the foundation for further development. In this research, we compared risk estimation for the HMM-based model to the previously published BN model.

### Statistical analysis

Baseline demographic and clinical characteristics were expressed as the means ± standard deviations and frequencies (percentages) based on normality and the continuous nature of the variable. All statistical analyses were conducted using R software (version 3.6.0, http://www.Rproject.org).

## Results

### Patient characteristics

**Figure 2:**
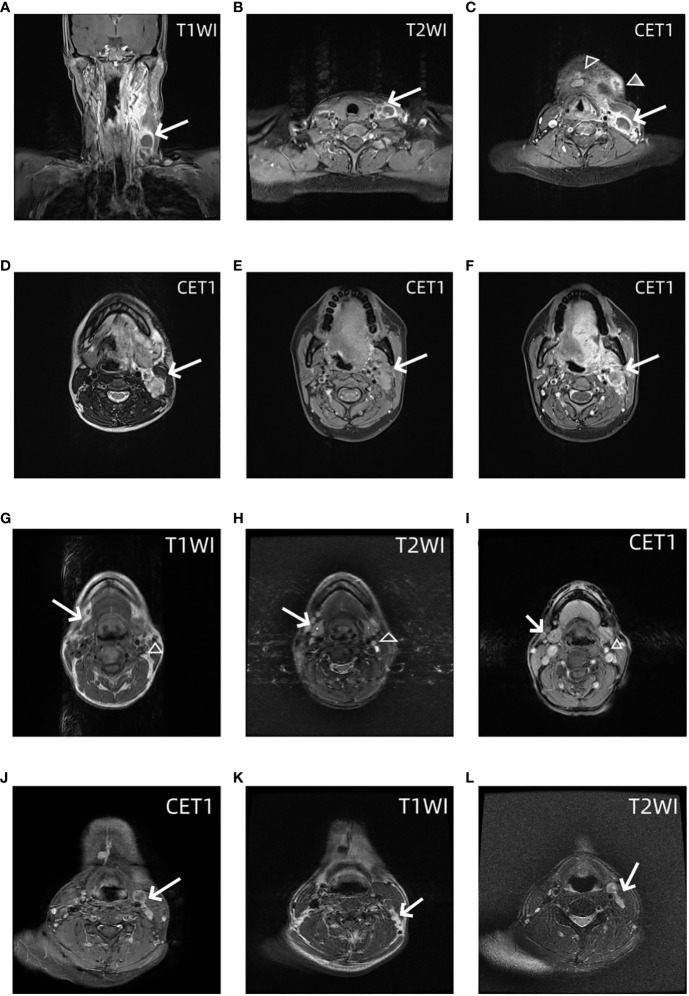
MRI images of the lower neck of a patient with a T4 stage OTSCC. **(A)** Coronal contrast enhanced T1 weighted images shows Levels III and IV lymph nodes have macroscopic metastasis (arrow). **(B)** Axial contrast enhanced T1 weighted images show Levels IV lymph nodes have macroscopic metastasis (arrow). **(C)** Axial contrast enhanced T1 weighted images show Levels I (trangle) and III lymph nodes (arrow) have macroscopic metastasis. MRI images of the upper neck of the same patient. Axial T1 weighted images **(D)** and T2 weighted images **(E)** and contrast enhanced T1 weighted images **(F)** show Levels II lymph nodes have macroscopic metastasis (arrow). MRI images of the neck of OTSCC patients with stage T3 occult lymph node metastasis. Axial T1-weighted images **(G)**, T2-weighted images **(H)**, and T1-enhanced images **(I)** show grade I and grade II occult lymph node metastases (empty triangle and arrow), that is, normal lymph nodes were diagnosed on MRI, but lymph node metastases were found on pathology. In another patient, axial T1 enhanced images **(J)**, T1-weighted images **(K)**, and T2-weighted images **(L)** show grade III and IV occult lymph node metastases (empty triangle and arrow).

There were 108 eligible patients enrolled in this study according to our criteria. Level I–IV lymph nodes were evaluated based on MRI. One representative analysis of LNLs is presented in **Figure 2**. A total of 79 patients were men, and 29 patients were women. The average age of the tongue cancer patients at diagnosis was 49.66 ± 12.77. There were 20 of 108 (19.44%) patients who had a history of chewing areca nuts. There were 66 of 108 (55.56%) patients who were in the early stage, and 48 (44.44%) patients were in the late stage. Details on the patient characteristics as well as the data available are provided in **Table 1**.

**Table 1:** Selected patient, tumor, demographic, and clinical characteristics of patients with tongue squamous cell carcinoma seen at Nanfang Hospital from 2016 to 2023.

Variable			Stratification
Age (y)			49.66 ± 12.77
Sex	Men	79	73.15%
	Women	29	26.85%
Areca nut history		21	19.44%
T stage	I	20	18.52%
	II	40	37.04%
	III	30	27.78%
	IV	18	16.67%

### Estimated subclinical rates

**Figure 3:**
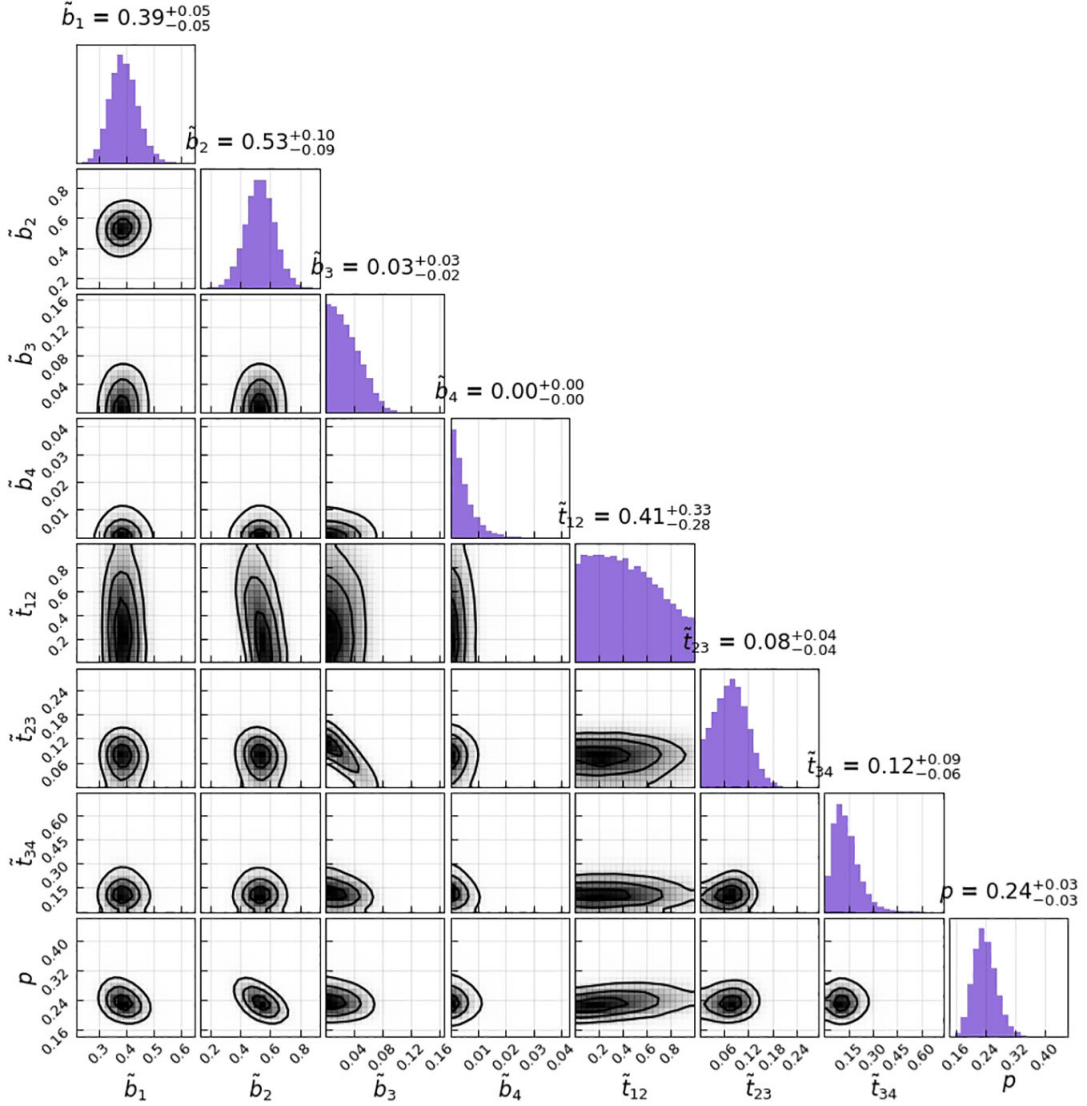
Corner plot of the sampled parameters for the HMM model parameters. The histograms on the diagonal show the 1D marginals, whereas the lower triangle shows all possible combinations of 2D marginals; the black lines are the isolines enclosing 20%, 50%, and 80% of the sampled points; correlations between the parameters can at most be seen between t12 and b2.

In our case, the starting state corresponds to a primary tumor being present, but all LNLs are still in the healthy state. The observation matrix (**Figure 3**) reflected the direct spread of tumor cells from the primary tumor to the LNL. Our results reflected that the most common involvement was that of level I and level II, corresponding to a high probability *p*b1=0.39±0.05, *p*b2= $0.53_{-0.09}^{+0.10}$ for the tumor to spread to level I and level II. Involvement of levels III and IV was gradually less common, corresponding to lower values of *p*b3= $0.03_{-0.02}^{+0.03}$ and *p*b4=0.00±0.00.

**Figure 4:**
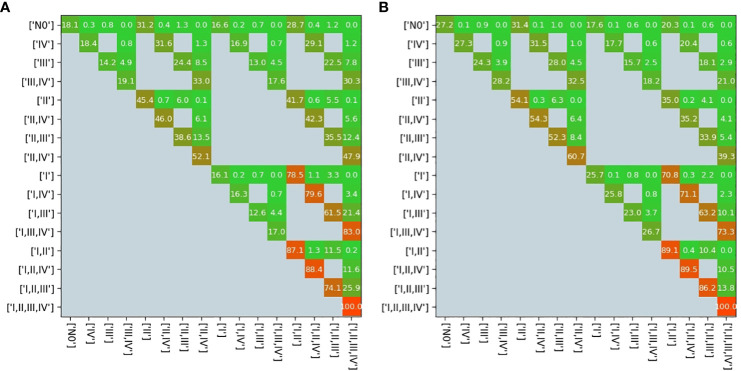
Transition matrix in the early stage **(A)** and in the late stage **(B)**. All gray pixels in this image correspond to entries in the matrix being zero; the colored pixels take on values ∈ [0,1], which are overlaid here in %; the exact values represent transition probabilities.

**Figure 5:**
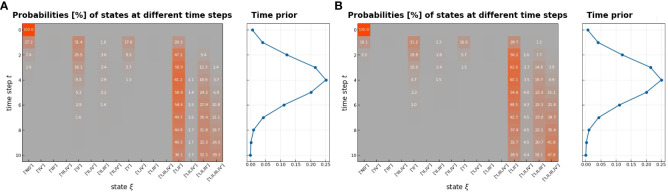
Probability of being in each hidden state as a function of time (left), early stage **(A)**, and late stage **(B)**; the color indicates low (green) and high (red) probabilities, which are also written on the respective pixel in percent if larger than 1%; we used the mean of the inferred parameter samples to compute the probabilities; on the right, the used time-prior is plotted with which each column on the left will be weighted.

The transition matrix reflected that the probability of a state transformation was correlated between the present state of metastases in lymph nodes and its timing (**Figure 4**). Our model estimated the risk of microscopic involvement for levels I and II simultaneously as 70.8% (**Figure 4B**) for the early stage patients (T1–T2) and 78.5% (**Figure 4A**) for the late-stage patients (T3–T4). If only level I was macroscopically involved, the risk of microscopic involvement for levels I, II, and IV simultaneously was 71.1% for the early-stage patients. If only levels I and IV were macroscopically involved, the risk was 79.6% for the late-stage patients. The risk of microscopic involvement for levels I, II, and III simultaneously was 63.2% for the early-stage patients and 61.5% for the late-stage patients. If levels I and III were macroscopically involved, the risk of microscopic involvement for levels I, II, III, and IV simultaneously was 73.3% for the early-stage patients. For late-stage patients, if only levels I, III, and IV were macroscopically involved, the risk was 83%. We chose a time prior for parametric learning. The binomial distribution had an intuitive shape and simple structure. Then, we could model how the state of an LNL involvement evolved over the time steps that support the chosen prior. In **Figure 5**, we depict the probability of each hidden state for each time step (calculated for the mean over all parameter samples). At time-step 0, the patient is healthy, and the system is in the initial state with probability 1. One time step later, the individual lymph nodes are involved with the base probability rates (**Figure 5**). The strongest correlations between the parameters can be seen between t12 and b2, which is consistent with the result presented in **Figure 3**.

**Figure 6:**
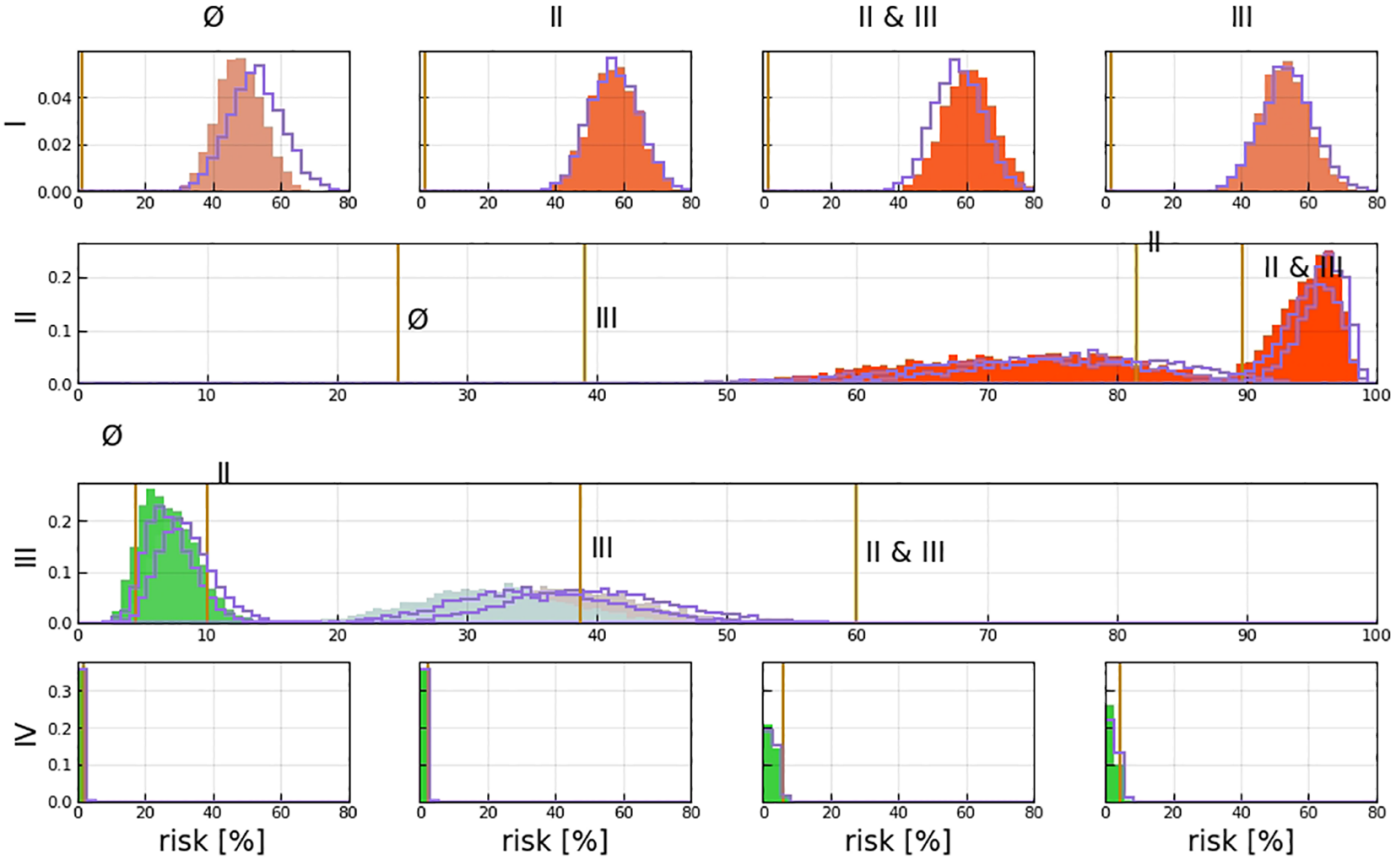
Risk assessment for the involvement of different LNLs (rows), given positive observational findings in specified LNLs (columns or labels next to histograms). For example, row 3 depicts the risk of involvement in LNL III, given different observed involvements (from left to right: no involvement, LNL II only, LNL III only, and LNL II and III but no others); the orange line depicts the maximum likelihood result from Pouymayou et al. (11), the violet outline histogram represents the BN sampling solutions, and the solid-colored histograms are the results from the HMM; the color goes from green (low risk) to red (high risk).

**Figure 6** shows the estimated risk of involvement for four possible observational states. LNL II and III account for the majority of level II involvement (≈95%). Involvement in level II further increases the risk for metastases in level III to almost 1% since the main cause of LNL III’s involvement is the spread from an already involved level II. Finally, the risk of involvement in level IV is increased from 0.78% to 2.28% and 2.75% when observing metastases in level III or in both level II and III, respectively (**Table 2**). **Figure 6** and **Table 2** also compares risk estimation for the HMM-based model to the BN model. The parameters of the BN model have been sampled from the likelihood function. The histograms of estimated risk are nearly identical, which verifies that the HMM-based model and the BN-based model yield the same risk predictions.

**Table 2:** Comparison among the estimated risks by the HMM (first line), the risk by the BN model (second line), and max likelihood model reported by Sanguineti et al. (third line) (11).

	Macroscopically involved LN levels:			
Probability (%) of microscopic involvement at level	N0	II only	II and III	III only
I	48.04	57.34	60.81	52.79
	52.85	57.40	57.63	53.78
	1.53	1.64	1.66	1.56
II	68.40	93.79	95.40	74.74
	72.08	94.71	95.85	76.79
	24.67	81.55	89.64	39.06
III	6.17	7.88	37.82	31.88
	7.18	8.46	39.61	35.43
	4.48	9.97	59.93	38.75
IV	0.78	0.95	2.75	2.28
	0.96	1.03	2.84	2.60
	1.83	2.25	6.05	4.44

**Figure 7:**
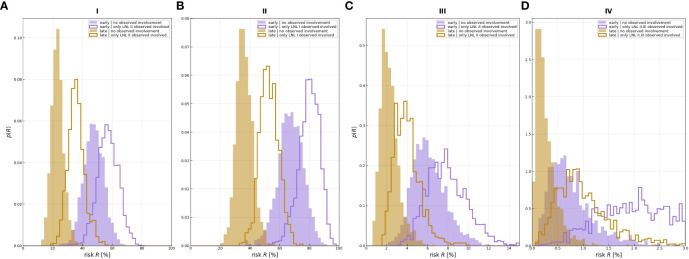
Distributions over risk of involvement for LNL I **(A)**, LNL II **(B)**, LNL III **(C)**, and LNL IV **(D)**, each for early and late T-staging as well as depending on the given observed involvement. The sampled parameters displayed here are a randomly selected subset from learning.

A comparison of the involvement risk for LNLs I–IV for early and late T stages given different observed prior diagnoses is shown in **Figure 7**. When only level II is observed to harbor metastases, the risk of microscopic involvement in level III increased approximately 2% in the early stage. If, in addition, the patient has a late T-stage tumor, the risk decreased by approximately 4%. Similarly, the risk of microscopic involvement in level IV for early and late T-stage patients increased by approximately 0.1% when levels II and III are observed to harbor metastases. If, in addition, the patient has a late T-stage tumor, the risk decreased approximately 0.5%. According to our data, the probability of developing level I and II lymph node metastases directly from a tumor is high (20% and 40%). When one macroscopic level is involved, microscopic involvement of levels I and II for early and late T-stage patients increased by approximately 20% and in the late stage, the risk decreased by approximately 40%.

**Figure 8:**
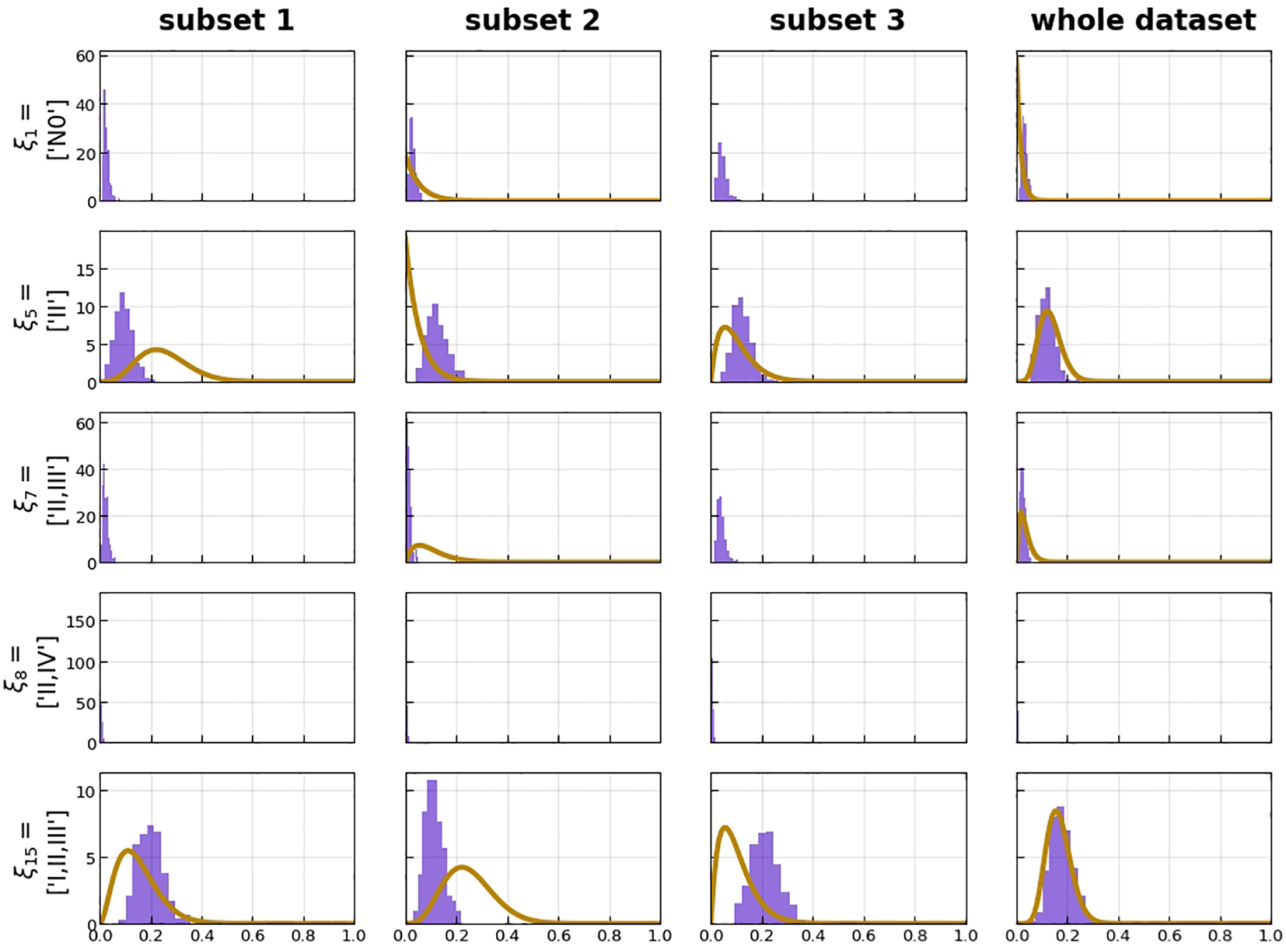
Histograms over predicted risk of certain states (violet) compared with the beta distribution over the same risk, resulting from the prevalence of the respective state in the dataset (orange). This is plotted for the three subsets of the threefold cross-validation as well as the whole dataset.

Predicted risk of certain states compared with the beta distribution over the same risk, resulting from the prevalence of the respective state in the dataset, showed that our model had good performance with a likelihood of −486.81 of the whole data set (**Figure 8**).

## Discussion

Oral cancer is a prevalent malignant tumor, with over 400,000 new cases detected globally each year. SCCs are the most common form, accounting for approximately 90% of malignant tumors in the oral region. Among the anatomical subsites of the oral cavity, the highest incidence of SCCs occurs in the oral tongue (35.3%) (12–14). The prognosis of oral SCCs patients depends on tumor and host-related characteristics as well as variations in treatment. The N stage is considered a crucial predictive factor, as the presence of a single metastatic lymph node in the regional lymph basin at referral can result in a 50% decrease in the 5-year survival rate (15). SCCs have a high potential for spreading to cervical lymph nodes. Regional lymphatic drainage pathways are well identified in the head and neck region, forming the basis for some revisions in neck dissection. For SCCs of the oral tongue, the submental, submandibular, and upper jugular lymph nodes are in the first echelon draining lymph nodes.

Level of metastatic lymph nodes (LLN) are recognized as an unstable lymph node group, necessitating surgical treatment expansion for LLN metastasis, especially in tongue cancer patients. The anatomically acquired incidence of LLN ranges from 8.6% to 30.2% (16). This requires various preoperative imaging examination modalities followed by meticulous data analysis. Both CT and MRI have been shown to be effective in imaging LLN metastasis in N+-stage patients (17).

CT and MRI are increasingly commonly utilized for preoperative assessment of primary tumors because of their better anatomical resolution. Enhanced CT and MRI may also be utilized to evaluate the condition of the lymph nodes if the primary tumor is determined to be the cause (18–20). Among the main variables used in traditional imaging procedures in determining probable lymph node metastases are lymph node size, contrast enhancement, and the existence of central necrosis or extranodal extension. Sadly, diverse or insufficiently stated successful tests prohibit accurate meta-analysis between studies. Depending on whether sensitivity as well as specificity was favored, additional criteria might be applied. Although the use of imaging techniques in a clinical suspicion context has proven valuable, a study by Chen et al. (21) suggests that routine monitoring imaging examinations for asymptomatic patients should not be encouraged. We proposed to use an HMM model for lymphatic metastatic spread that, given the location of macroscopic metastases and T-stage, can quantify the risk of microscopic, infiltrated LNLs and their changes continuously over time. Here, we demonstrated an HMM can predict the probability of individual lymph node metastasis and its changes over time.

Based on our series of 108 patients with histologically proved OTSCCs, we found that for N0 patients, neck levels I and II were at the greatest risk of nodal metastases from primary squamous cell carcinoma of the oral cavity (0.39, 0.53). Additionally, our data showed that lymph node levels I and II were the most common patterns and had strong correlations with other lymph node levels (levels III and IV) involvement. For an example, when lymph nodes I and II have metastasis, other lymph node metastases are highly likely to occur in the near future. In NCCN guidelines, selective neck dissections are based on common pathways of the spread of head and neck cancer to regional nodes and are often recommended for N0 disease (22).

According to our results, neck level I and II dissections would benefit N0 disease patients. Our findings also indicated that when lymph node level I had metastasis, the probability of metastasis in lymph node II was high (93.79%); when lymph node level II had metastasis, the probability of metastasis in lymph node III was small (7.88%), as well as the probability of involvement in lymph node IV. Based on this finding, prophylactic level I and level II excision should be considered for those with level I metastasis in N1–2 individuals. Furthermore, in patients with level II metastases, preventive level I and level II resection is favored over unnecessary level III excision. Our results also suggest that, when other lymph node levels were metastasized, lymph node level II had the highest likelihood of invasion. Therefore, based on these findings, we suggest the removal of lymph node level II when other levels of lymph nodes metastasize. The involvement of lymph nodes I and II was the most common form and showed a binomial distribution over time.

Moreover, we found that lymph nodes progress faster in the early stage and slower in the late stage, which needs to be confirmed in a larger clinical trial in the future. It could be that the disease advances faster in the early stages while progressing more slowly in the later phases, or that it is more likely to spread to higher-grade lymph nodes, which would explain the observed behaviors. Lymph node metastases, for instance, have generally occurred at levels 5–level 8 rather than levels 1–4. A notable innovation in our work was the introduction of additional time points, and instead of waiting for signs of lymph node involvement, we can remove the suspect lymph node beforehand or administer radiation therapy.

This treatment method, selective neck resection, may occur when latent metastasis progresses to clinically severe disease, when patients are diagnosed with incurable neck disease, or when undiagnosed metastatic lymph nodes advance toward a detectable size.

Of note, head and neck cancer could be linked to an increased risk of lymph node metastasis when left ignored. Although there may be an increase risk in head and neck cancers when cervical lymph node disease is untreated, some reasons support withholding cervical lymph node treatment. A significant portion of patients experience a decrease in quality of life (shoulder dysfunction, for example) and needless medical expenses (22–24). Additionally, the cancer growth could be effected by this additional treatment. Nevertheless, there might be fewer surgical problems, as well as good functional and superficial results with selective neck dissection. If cN0 is not treated electively, close follow-up with the obligatory use of diagnostic techniques is done. Diagnostic methods like ultrasound-guided FNA cytology have to be utilized during surgical follow-up in order to identify latent metastases early on. Most patients can avoid unnecessary selective neck resection with this approach, all without sacrificing regional control or neck survival. Nonetheless, neck treatment will be more involved and particularly frequent for those who finally require a (salvaging) lymph node dissection as a result of delayed metastases. The validity of clinical N0 classification depends on the diagnostic method used. Studies that apply negative palpation to define N0 neck may have a higher sensitivity than those that apply a more sophisticated assessment with prospective staging techniques. The method of MRI examination was adopted in this study (23, 25).

The risk of microscopic lymph node involvement not only changes over time in relation to the location of macroscopic metastases found with imaging but is also associated with the T stage. This is one advantage of HMM. Pouymayou et al. (11) presented a comprehensive model of microscopic lymph node involvement in HNSCC based on a Bayesian network. The model provides a statistical framework that combines the probabilities of direct infiltration from the primary tumor site, the spread through the lymphatic network, and the specificity and sensitivity of current diagnostic methods. However, our study further confirmed that a multi-time-point model outperformed the single-time-point BN model in predicting lymph node involvement. Esce et al. (26) reported that a CNN can produce an algorithm capable of predicting nodal metastases in patients with squamous cell carcinoma of the oral tongue by analyzing the imagological examination of the primary tumor alone. However, it may be insufficient to evaluate and predict individual progression status based on a single time point.

Due to the retrospective nature of our study, there are some limitations. Learning the model parameters must be based on a larger training dataset of lymph node progression patterns, including additional LNLs, such as levels V–VII. Due to the rarity of these levels, a larger training dataset is needed. It is also necessary to consider ipsilateral spread, as well as considering patient-specific observations such as midline extension of the primary tumor. Here, one might also expect the transfer probabilities between ipsilateral and contralateral lymph node metastases to remain similar since clinically managed lymph node metastases are bilateral. However, this study did not separately establish a model for ipsilateral lymph node metastasis. Furthermore, we would like to include other risk factors, such as HPV status, age, alcohol, and nicotine abuse, in the model at some point in the future. MRI sensitivity and specificity can vary depending on the device utilized, the radiologist’s experience, and the unique features of the patient. The sensitivity and specificity of the MRI images utilized in this research were determined based upon a 2007 meta-analysis. There were 1.16% and 59.30% overall false positives as well as false negatives at our institution’s MRI examinations. Following studies will employ a spectrum of values to account for this fluctuation and offer a more sophisticated evaluation, offering additional insight into the factors influencing the model’s prediction.

Lymph node metastasis in the neck directly impacts treatment and outcomes. Single lymph node metastasis from HNSCC reduces survival by 50%, whereas additional contralateral lymph node metastasis reduces survival to 33% (27). Expected drainage sites and oral tongue malignancies drain to levels I and II (28, 29). However, skip metastasis also occurs with rates ranging from 6% to 10% (30). The odds for primary location metastases for levels III and IV of the research we conducted were 6.17% and 0.78, respectively. Contralateral lymph node metastasis is also observed in tongue malignancies due to disease crossing the midline. Imaging studies aid in detecting occult metastases not apparent in clinical examinations. The proposed method can be used to guide guidelines for selective lymph node CTV matching or surgical excision range. By incorporating the T category as an additional risk factor, this method extends the previous estimation of microscopic involvement risk at the lymph node level. When provided with a larger, more diverse dataset, this model may support clinicians in making CTV-N defense more objective and personalized.

## Conclusion

We applied an HMM probability model to predict the progression of lymphatic tumors over time, extending previous work to estimate the risk of microscopic metastasis at the lymph node level by including T-stage as an additional risk factor. The BN model and max likelihood model reported previously were compared with our results. This model can predict lymph nodes that are likely to be involved in the future ahead of clinical and imaging findings and can support clinicians in prophylactic excision or radiation therapy.

## Data availability statement

The raw data supporting the conclusions of this article will be made available by the authors, without undue reservation.

## Ethics statement

The studies involving humans were approved by Nanfang Hospital, Southern Medical University. The studies were conducted in accordance with the local legislation and institutional requirements. Written informed consent for participation was not required from the participants or the participants’ legal guardians/next of kin in accordance with the national legislation and institutional requirements.

## Author contributions

QG: Conceptualization, Data curation, Formal analysis, Methodology, Software, Visualization, Writing – original draft. JF: Data curation, Supervision, Writing – review & editing. H-UK: Supervision, Writing – review & editing. KZ: Project administration, Supervision, Writing – review & editing.
