# Correction: Predicting nodal metastasis progression of oral tongue cancer using a hidden Markov model in MRI

**DOI:** 10.3389/fonc.2026.1877999

**Published:** 2026-05-26

**Authors:** Qiangqiang Gang, Jie Feng, Hans-Ulrich Kauczor, Ke Zhang

**Affiliations:** 1 Department of Radiology, Southern Medical University Nanfang Hospital, Guangzhou, China; 2 Department of Diagnostic and Interventional Radiology, Heidelberg University Hospital, Heidelberg, Germany

**Keywords:** artificial intelligence, hidden Markov model, level of metastatic lymph nodes, lymph node metastases, oral cavity squamous cell carcinoma

“Ludwig R, Pouymayou B, Balermpas P, Unkelbach J. A hidden Markov model for lymphatic tumor progression in the head and neck. Scientific Reports. 2021;11(1):12261” was not cited in the article. The citation should be inserted in the section **Materials and Methods**, subsection *Model Training and Validation*, after the sentence “An HMM was used to compute the probabilities of transitions between states over time subsection” and should read: “An HMM was used to compute the probabilities of transitions between states over time (10)”.

The original version of this article has been updated.

